# Supplementary material for: Efficient generation of marmoset primordial germ cell-like cells using induced pluripotent stem cells
Source: eLife. 2023 Jan 31;12:e82263. doi: 10.7554/eLife.82263 (PMC9937652; doi:10.7554/eLife.82263)
Supplement: Figure 1—source data 1. [file elife-82263-fig1-data1.pdf]

Figure 1–Source Data 1

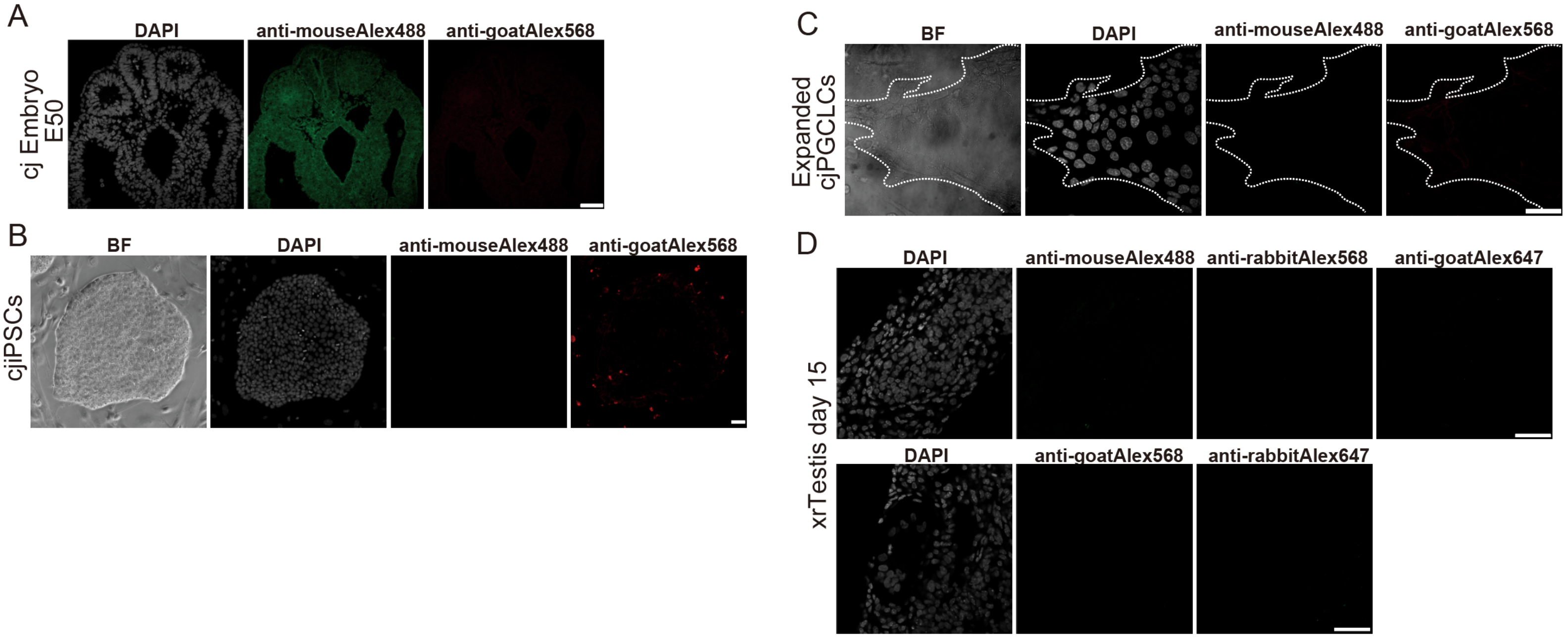

Figure 1–Source Data 1. Negative control images for immunofluorescence studies.
(**A**) IF images of a cj embryo at E50 for DAPI (white), Alexa Fluor 488 conjugated donkey anti-mouse IgG (green) and Alexa Fluor 568 conjugated donkey anti-goat IgG (red). A section adjacent to one used in Figure 1C was used. Primary antibodies were not added. Scale bar, 50 μm.
(**B**) IF images of cjiPSCs (OF/IWR1), related to Figure 3–figure supplement 2E, for DAPI (white), Alexa Fluor 488 conjugated donkey anti-mouse IgG (green) and Alexa Fluor 568 conjugated donkey anti-goat IgG (red). Primary antibodies were not added. Scale bar, 50 μm.
(**C**) Bright field and IF images of expansion culture c10 cjPGCLCs, related to Figure 4D, for DAPI (white), Alexa Fluor 488 conjugated donkey anti-mouse IgG (green) and Alexa Fluor 568 conjugated donkey anti-goat IgG (red). Primary antibodies were not added. Scale bar, 50 μm.
(**D**) (top) IF images of a xrTestis at day 15, related to Figure 5C, for DAPI (white), Alexa Fluor 488 conjugated donkey anti-mouse IgG (green), Alexa Fluor 568 conjugated donkey anti-rabbit IgG (red) and Alexa Fluor 647conjugated donkey anti-goat IgG (cyan). (bottom) IF images of a xrTestis at day 15 for DAPI (white), Alexa Fluor 568 conjugated donkey anti-goat IgG (red) and Alexa Fluor 647 conjugated donkey anti-rabbit IgG (cyan). Primary antibodies were not added. Scale bar, 50 μm.
