# Supplementary material for: Efficient generation of marmoset primordial germ cell-like cells using induced pluripotent stem cells
Source: eLife. 2023 Jan 31;12:e82263. doi: 10.7554/eLife.82263 (PMC9937652; doi:10.7554/eLife.82263)
Supplement: Supplementary file 1. [file elife-82263-supp1.docx]

**Supplementary File 1. Primers used in this study.**

| **Gene Symbol**  **(*Callithrix jacchus*)** | **Forward primer (5’ 🡪3’)** | **Reverse primer (5’ 🡪3’)** |
| --- | --- | --- |
| *GAPDH* | AATTTGGCTACAGCAACAGGGT | GAGTGAGGGCCTCTTTTCCTC |
| *PPIA* | CATTGCTGACTGTGGACAACTT | CTGCAGCGAGAGCAGATTTTAG |
| *NANOG* | CCAGTCAATCTGAAAGGAGGGT | GGTGTCCTTAGTAGCCAAGGTT |
| *SOX2* | TCCCAACTTTCCATTTTGTTCAGA | CAGCTACGGAAAATAAAGGGGG |
| *POU5F1* | TCAAGCAGTGACTATGCACAAC | ATAGCCTGGGGTACCAAAATGG |
| *UTF1* | CGACATCGTGGGTATCCTGG | GGACACGGTCTGGTCGAAGG |
| *LIN28A* | TATGGGAGGGAGGGTAGGAAAG | CCATCAAATAAACGCACCCCAG |
| *TBXT* | GGCTGGGGACCCAGTTCTTC | TTCGTACAGTGGGGATCCAGAG |
| *EOMES* | GGCAAGGGGAGCATTTCATCA | GCGCAGGAAGATGAAATAGGAG |
| *MIXL1* | TCATCCTTAGCCTGCAGAACAG | CAGGGGAGTCCTCAAGTGTTTT |
| *GATA4* | GGACTCTGTCCTCGTCCTCTTT | AGGATTTTGGAGTTAGGGGCTT |
| *SOX17* | CCCTGGAATGACCCTACTTCAT | CTGGACGTCCCCTTCTCATTTA |
| *TFAP2C* | GGGGTAATCTCTAAAGCACCGT | TCTTCCAAGCCAAGAACAGAGC |
| *PRDM1* | CACCATCCATCCTTCTTTTCCG | AGATAGTTCGTTGTTCTAGCAAAGT |
| *NANOS3* | AGGTCACCTTAGGAGGATCG | TGTTCCTTTGACAACCCCAGAC |
| *DPPA3* | AGCTGTACTGTTTGGTTTGTCC | TCAGTGTCTTGTTGCTGGGTT |
| *DDX4* | TGATGAGTCATGGGATTAAAACTGAA | CACAAGGACAGGAGCTATGAC |
| *DAZL* | CCGCTGTCTAGTTTCATGGGAA | GCTTATGCAAGAAGTTTGGTGTG |
| *ITGA6* | AGGATGTTATGGTGTGTACAGTT | TGCTAGTTAGTATAAAAATGTATCAAATGC |
| *FOXA2* | CTGTTGCTGCAGGGAAGTCTTA | AGTACAACCCTCTGGTTCTGTT |
| *AFP* | TTGCAGGCATGTTGGAGAAATG | TCCCCCTGTCATTTAAACTCCC |
| *SOX1* | TAATTGTCCTGGAGCGAGTACG | GCTGTTCTCAGATGTAGGCTGA |
| *PAX6* | CCTGGAACAACATGCACTAGAT | ACACTCTACCTTTTAGCTATCAACT |
| *PDX1* | CAAGGGAAAACTACCCAACCCA | TAAACTTAAAGTGGTCGCCGGA |
| *CXCR4* | TGTCTGGTGGTAGGACCGTAG | AAAGAAAGAGATGTTCCACGGG |
| *SOX7* | CCTTTTTGTCCTTCCAAGACATCC | CGCCCGTCAGTCATATCATCAA |
| *GATA6* | TCTCAGGAAAATTGCCTTTCTCT | TTGTTGCAATTTTTCCAGCACA |
| *MSX1* | CAAGCCAGGGAGATGAATCCT | CAACCTACCTTTGCAATCGTCT |
| *HAND1* | CCAGACGTAGGAAGATGAAGGG | GAACTAAACAGGAAGTGCAGCG |
| *DLK1* | AGATCGGCATGGCCACCTTC | CTAGCGAACTCCACCACAAAGA |
| *LHX2* | CTGCCACGTGCCTTAGGAATA | TCATAAATATACAGAGAGAAAAAGAGGGA |
| *NES* | GTCAGCTGGAAAGTCTGGGAC | TCCATTAGCCACAGGCCAGA |
| *OTX2* | CTGGACGAGGAGAGTGAGAGAA | AGTAATCCATCAGGGTCAGGGC |
| *NCAM1* | ATAGTGCAGCTTTGGAAGTGGA | AAAACCACCATCCAGACAGACA |
| *RAX* | CCCTATCTCTCTGGAAGGTGGA | TATTTCGGGTAAAGGGGAACCG |
| *HES5* | CTTTTGTGAAGGCCGAACTCAA | CATTCAGAGCCTTTTGGCCACG |
